# Whole genome shotgun sequence of *Bacillus amyloliquefaciens* TF28, a biocontrol entophytic bacterium

**DOI:** 10.1186/s40793-016-0182-6

**Published:** 2016-09-21

**Authors:** Shumei Zhang, Wei Jiang, Jing Li, Liqiang Meng, Xu Cao, Jihua Hu, Yushuai Liu, Jingyu Chen, Changqing Sha

**Affiliations:** 1 Institute of Microbiology, Heilongjiang Academy of Sciences, Harbin, 150010 China; 2 Institute of Advanced Technology, Heilongjiang Academy of Sciences, Harbin, 150020 China; 3 Heilongjiang Academy of Sciences, Harbin, 150001 China

**Keywords:** Genome sequence, *Bacillus amyloliquefaciens*, Endophytic bacterium, Biocontrol, Broad spectrum

## Abstract

*Bacillus amyloliquefaciens* TF28 is a biocontrol endophytic bacterium that is capable of inhibition of a broad range of plant pathogenic fungi. The strain has the potential to be developed into a biocontrol agent for use in agriculture. Here we report the whole-genome shotgun sequence of the strain. The genome size of *B. amyloliquefaciens* TF28 is 3,987,635 bp which consists of 3754 protein-coding genes, 65 tandem repeat sequences, 47 minisatellite DNA, 2 microsatellite DNA, 63 tRNA, 7rRNA, 6 sRNA, 3 prophage and CRISPR domains.

**Electronic supplementary material:**

The online version of this article (doi:10.1186/s40793-016-0182-6) contains supplementary material, which is available to authorized users.

## Introduction

*Bacillus amyloliquefaciens* is ubiquitous in nature. Some strains are used as biocontrol agents because of their ability to produce antagonistic metabolites, plant growth promoters and plant health enhancers [1–4]. *B. amyloliquefaciens* is usually divided into two subspecies by genome comparison and classical bacterial taxonomy. Plant growth-promoting rhizobacterial strains are classified as *B. amyloliquefaciens subsp. plantarum*, while other strains are regarded as *B. amyloliquefaciens subsp. amyloliquefaciens* [5]. *B. amyloliquefaciens* TF28 is an endophytic bacterium that was isolated from soybean root. Previous studies have shown that *B. amyloliquefaciens* TF28 could inhibit soil borne and air borne plant pathogenic fungi by competition, synthesizing antifungal metabolites and inducing systemic plant resistance [6, 7]. Based on 16S rRNA, DNA gyrase subunit A (*gyrA*) and RNA polymerase subunit B (*rpoB*) gene sequence analysis, *B. amyloliquefaciens* TF28 was classified as *B. amyloliquefaciens subsp. plantarum*. Here we present a whole-genome shotgun sequence of *B. amyloliquefaciens* TF28 and its annotation for facilitating its application in the biocontrol of plant diseases.

## Organism information

### Classification and features

**Fig. 1:**
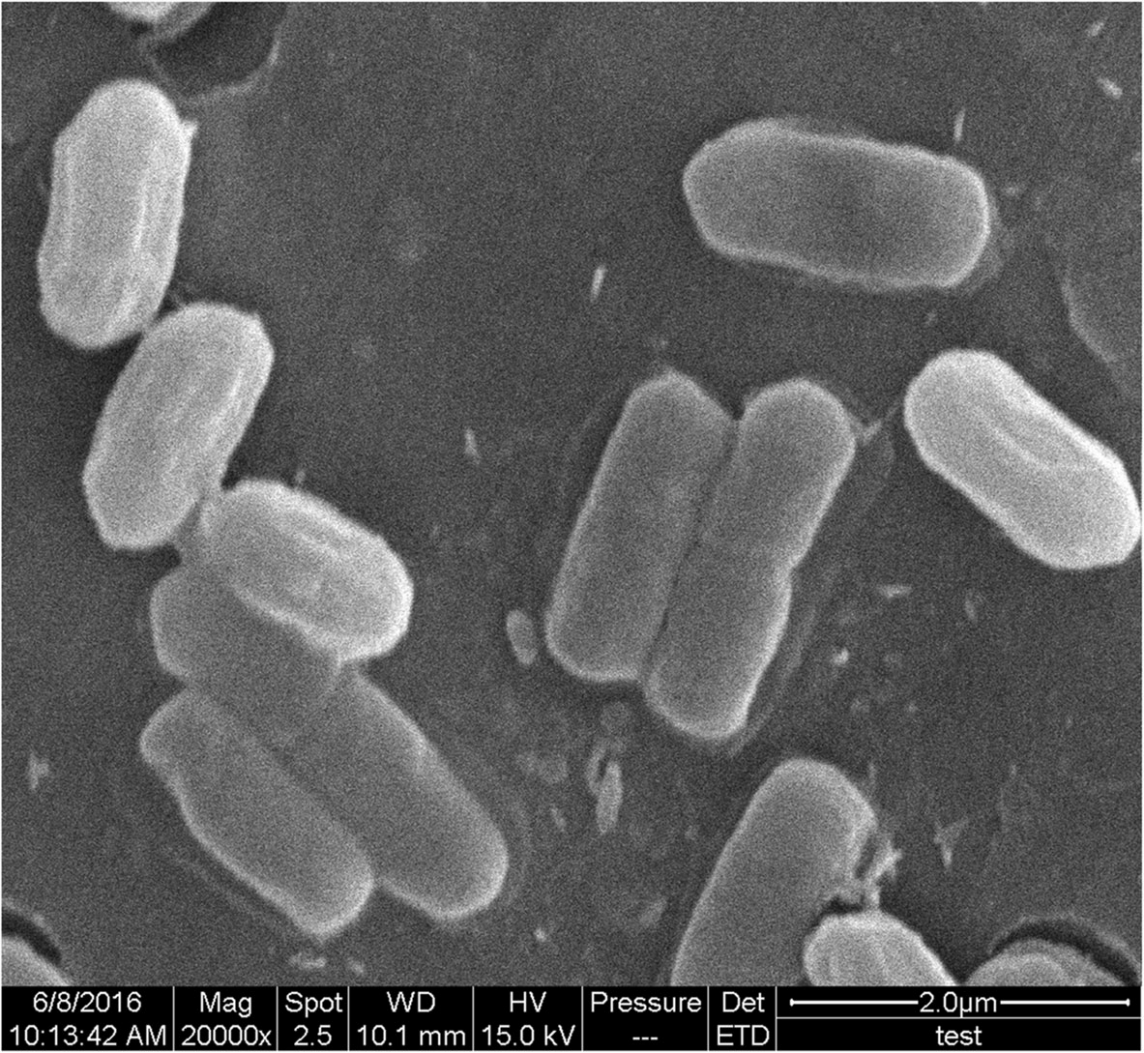
A scanning electron micrograph of *B. amyloliquefaciens* TF 28 cells

*B. amyloliquefaciens* TF28 was isolated from soybean root in China. It exhibited an unusual ability to inhibit a wide range of plant pathogenic fungi. The cell morphology of strain TF28 was determined using scanning electron microscopy (Fig. 1). Cells of *B. amyloliquefaciens* TF28 are Gram-positive, rod shape, aerobic and endospore- forming. Strain TF28 utilizes glucose and lactose to produce acid and hydrolyzed gelatin and starch. Starin TF28 is positive for Vogues-Proskaur and Methyl red reaction, nitrate reduction and citrate utilization. Current taxonomic classification and general features of *B. amyloliquefaciens* TF28 are provided in Table 1.

**Table 1:** Classification and general features of *B.amyloliquifaciens* TF28 as per MIGS recommendation [26]

MIGS ID	Property	Term	Evidence code^a^
	Classification	Domain *Bacteria*	TAS [27]
		Phylum *Firmicutes*	TAS [28–30]
		Class *Bacilli*	TAS [31, 32]
		Order *Bacillales*	TAS [29, 33]
		Family *Bacillaceae*	TAS [29, 34]
		Genus *Bacillus*	TAS [29, 35]
		Species *Bacillus amyloliquefaciens*	TAS [36–38]
		Strain: TF28	TAS [6]
	Gram stain	Positive	TAS [6]
	Cell shape	Rod	TAS [6]
	Motility	Motile	TAS [6]
	Sporulation	Endospore-forming	TAS [6]
	Temperature range	15–37 °C	TAS [6]
	Optimum temperature	30 °C	TAS [6]
	pH range; Optimum	5–9, 7.5	TAS [6]
	Carbon source	Glucose, lactose, starch	TAS [6]
MIGS-6	Habitat	Soil, Plant	TAS [6]
MIGS-6.3	Salinity	0–3 % W/V	TAS [6]
MIGS-22	Oxygen requirement	Aerobic	TAS [6]
MIGS-15	Biotic relationship	Free-living	TAS [6]
MIGS-14	Pathogenicity	Non-pathogen	NAS
MIGS-4	Geographic location	China/Heilongjiang	TAS [6]
MIGS-5	Sample collection	2006-06-10	TAS [6]
MIGS-4.1	Latitude	Not reported	
MIGS-4.2	Longitude	Not reported	
MIGS-4.4	Altitude	Not reported	

^a^Evidence codes - NAS: Non-traceable Author Statement (i.e., not directly observed for the living, isolated sample, but based on a generally accepted property for the species, or anecdotal evidence). These evidence codes are from the Gene Ontology project [39]

**Fig. 2:**
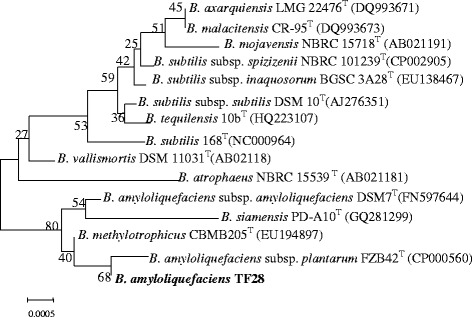
Phylogenetic trees based on 16S rRNA gene sequences highlighting the position of *B. amyloliquefaciens* TF 28 (shown in bold). The GenBank accession numbers are shown in parentheses. Sequences were aligned using CLUSTALW, and phylogenetic inferences were constructed using the neighbor-joining method within the MEGA 5.10 software (Additional file 7: Table S7). Numbers at the nodes represent percentages of bootstrap values obtained by repeating the analysis 1000 times to generate a majority consensus tree. The scale bar indicates 0.0005 nucleotide change per nucleotide position, respectively

The 16S rRNA gene sequence of strain TF28 and other available 16S rRNA gene sequences of closely related species collected from NCBI database were used to construct a phylogenetic tree (Fig. 2, Additional file 1: Table S1). The evolutionary history was inferred using the Neighbour-joining method with MEGA software version 5.10. BLAST analysis showed strain *B. amyloliquefaciens* TF28 shared 99.3–99.7 % 16S rRNA gene identities with the other 14 type strains of *Bacillus* species. Taxonomic analysis showed that 14 type strains were divided into two groups. Strain TF28 together with *B. amyloliquefaciens subsp. plantarum* FZB42^T^, *B. methylotrophicus* CBMB205^T^, *B. amyloliquefaciens* subsp. *amyloliquefaciens* DSM7^T^and *B. siamensis* PD-A10^T^ were clustered into one group. Other strains (*B. atrophaeus*NBRC 15539^T^, *B. vallismortis*DSM 11031^T^, *B. tequilensis* 10b^T^, *B. subtilis* 168^T^, *B. subtilis subsp. subtilis*DSM 10^T^, *B. subtilis subsp. inaquosorum*BGSC 3A28^T^, *B. subtilis subsp. spizizenii*NBRC 101239^T^, *B. mojavensis*NBRC 15718^T^, *B. malacitensis* CR-95^T^ and *B. axarquiensis*LMG 22476^T^) were clustered into another group. Two type stains of *B. amyloliquefaciens**sub*species, *B. amyloliquefaciens**B. amyloliquefaciens subsp. plantarum* FZB42^T^ and *B. amyloliquefaciens subsp. amyloliquefaciens*DSM7^T^ were attributed to the different clade. Strain TF28 was most closely related to *B. amyloliquefaciens subsp. plantarum* FZB42^T^ with 99.7 % 16S rRNA gene sequence identity. Strain TF28 was classified as *B. amyloliquefaciens subsp. plantarum*.

## Genome sequencing information

### Genome project history

Genome of *B. amyloliquefaciens* TF28 was sequenced by Huada Gene Technology Co., Ltd, Shenzhen, China. The Whole Genome Shotgun sequence has been deposited in GenBank database under the accession number JUDU00000000. The summary of the project information is shown in Table 2.

**Table 2:** Project information

MIGS ID	Property	Term
MIGS 31	Finishing quality	High-quality draft
MIGS-28	Libraries used	Illumina Paired-End Library (500 bp insert size) and Mate Pair Library (6,000 bp insert size)
MIGS 29	Sequencing platforms	Illumina Hiseq2000
MIGS 31.2	Fold coverage	150×
MIGS 30	Assemblers	SOAPdenovo software 2.04
MIGS 32	Gene calling method	Glimmer
	Locus Tag	TH57
	GenBank ID	JUDU00000000
	GenBank Date of Release	2015/01/21
	GOLD ID	-
	BIOPROJECT	PRJNA268537
MIGS 13	Source Material Identifier	TF28
	Project relevance	Biocontrol, Agriculture

### Growth conditions and genomic DNA preparation

*B. amyloliquefaciens* TF28 was grown in LB medium at 30 °C for 16 h. One liter cultures at the exponential growth phase was taken and centrifuged at 4 °C, 5000 rpm for 10 min. The pellet was collected and about 5 g cell pellet was used to extract genomic DNA by CTAB method [8]. The quality of DNA was assessed using a Qubit Fluorometer. Total DNA (280.6 μg) was obtained to do genome sequencing.

### Genome sequencing and assembly

Genomic DNA was sheared randomly. The required length DNA fragments were retained by electrophoresis and used for construction of a 500 bp and 6000 bp long paired-end library. Sequencing was performed by Illumina HiSeq 2000 sequencing platform. Sequencing of the 500 bp library generated 6,649,820 reads (representing 554 Mbp of sequence information), while sequencing of the 6,000 bp paired-end library generated 3,633,388 reads (290 Mbp). Both libraries achieved a genome coverage of 190× for an estimated genome size of 4.4 Mbp. All generated reads were quality trimmed to obtain clear reads. The trimmed reads were assembled by SOAPdenovo software 2.04 using the available genome sequence of *B. amyloliquefaciens subsp. plantarum* FZB42^T^(CP000560) as reference-guided assembling. The final assembly yielded 182 contigs and 3 scaffolds representing 3.9 Mbp of sequence information.

### Genome annotation

The genome sequence was annotated by a combination of several annotation tools. Genes were identified by Glimmer 3.02 [9]. DNA tandem repeat sequences, minisatellite DNA and microsatellite DNA were found with the Tandem Repeats Finder 4.04 [10]. Prediction of non-coding RNA was performed using rRNA database blasting or rRNAmmer 1.2 for rRNA [11], tRNAscan-SE 1.23 for tRNA and their secondary structure [12], and infernal software and Rfam database for sRNA [13]. Prophage was predicted using PHAST software 2013.03.20 [14]. CRISPR domains were found using CRISPR Finder 0.4 [15]. Functional annotation of protein coding genes was based on gene comparisons with GO database (version 1.419) [16], KEGG database (version 59) [17], Cluster of Orthologous Groups of proteins(COG)(version 20090331) [18], NR database(version 20121005), SwissProt (version 201206) [19] and Pfam databases (version 25) [20].

## Genome properties

**Fig. 3:**
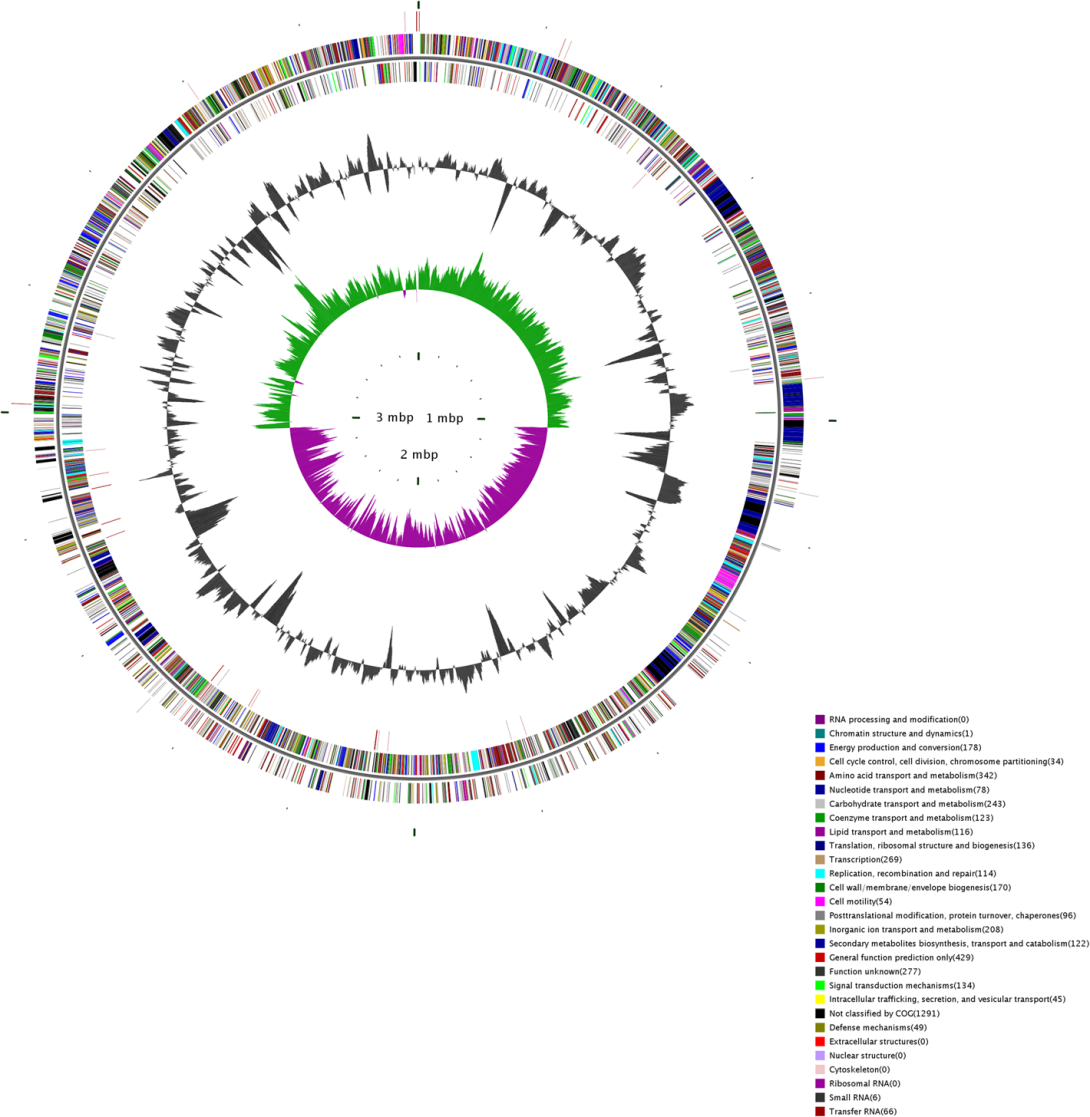
Circle map of strain TF28 genome. From outer to inner circle, circle 1 shows protein-coding genes colored by COG categories; circle 2 shows G + C% content plot; circle 3 shows GC skew

The genome statistics are provided in Table 3 and Fig. 3. The high quality draft genome of *B. amyloliquefaciens* TF28 was distributed in 182 contigs with a total size of 3,987,635 bp and an average G + C content of 46.38 %. Genome analysis showed that the genome of strain TF28 contained 3,754 protein coding genes, 65 tandem repeat sequences, 47 minisatellite DNA, 2 microsatellite DNA, 63 tRNA, 7 rRNA, 6 sRNA, 3 prophage and 3 CRISPR domains. The predicted protein coding genes represented 89.57 % of the total genome sequence, with a total length of 3,571,596 bp. The majority of protein coding genes (76.13 %) were assigned to putative functions. The distribution of genes into COG functional categories is presented in Table 4.

**Table 3:** Genome statistics

Attribute	Value	% of Total
Genome size (bp)	3,987,635	100.00
DNA coding (bp)	3,571,596	89.57
DNA G + C (bp)	1,849,465	46.38
DNA scaffolds	3	-
Total genes	3863	100.00
Protein coding genes	3754	97.18
RNA genes	76	1.97
Pseudo genes	38	0.98
Genes in internal clusters	1554	40.23
Genes with function prediction	2941	76.13
Genes assigned to COGs	3218	83.30
Genes with Pfam domains	3292	85.21
Genes with signal peptides	204	5.28
Genes with transmembrane helices	1041	26.95
CRISPR repeats	3	-

**Table 4:** Number of genes associated with general COG functional categories

Code	Value	%age	Description
J	136	3.02	Translation, ribosomal structure and biogenesis
A	0	0	RNA processing and modification
K	269	5.97	Transcription
L	114	2.53	Replication, recombination and repair
B	1	0.02	Chromatin structure and dynamics
D	34	0.75	Cell cycle control, Cell division, chromosome partitioning
V	49	1.09	Defense mechanisms
T	134	2.97	Signal transduction mechanisms
M	170	3.77	Cell wall/membrane biogenesis
N	54	1.19	Cell motility
U	45	0.99	Intracellular trafficking and secretion
O	96	2.13	Posttranslational modification, protein turnover, chaperones
C	178	3.95	Energy production and conversion
G	243	5.39	Carbohydrate transport and metabolism
E	342	7.58	Amino acid transport and metabolism
F	78	1.73	Nucleotide transport and metabolism
H	123	2.73	Coenzyme transport and metabolism
I	116	2.57	Lipid transport and metabolism
P	208	4.61	Inorganic ion transport and metabolism
Q	122	2.71	Secondary metabolites biosynthesis, transport and catabolism
R	429	9.51	General function prediction only
S	277	6.14	Function unknown
-	1291	28.63	Not in COGs

The total % age is based on the total number of protein coding genes in the annotated genome

## Insights from the genome sequence

**Fig. 4:**
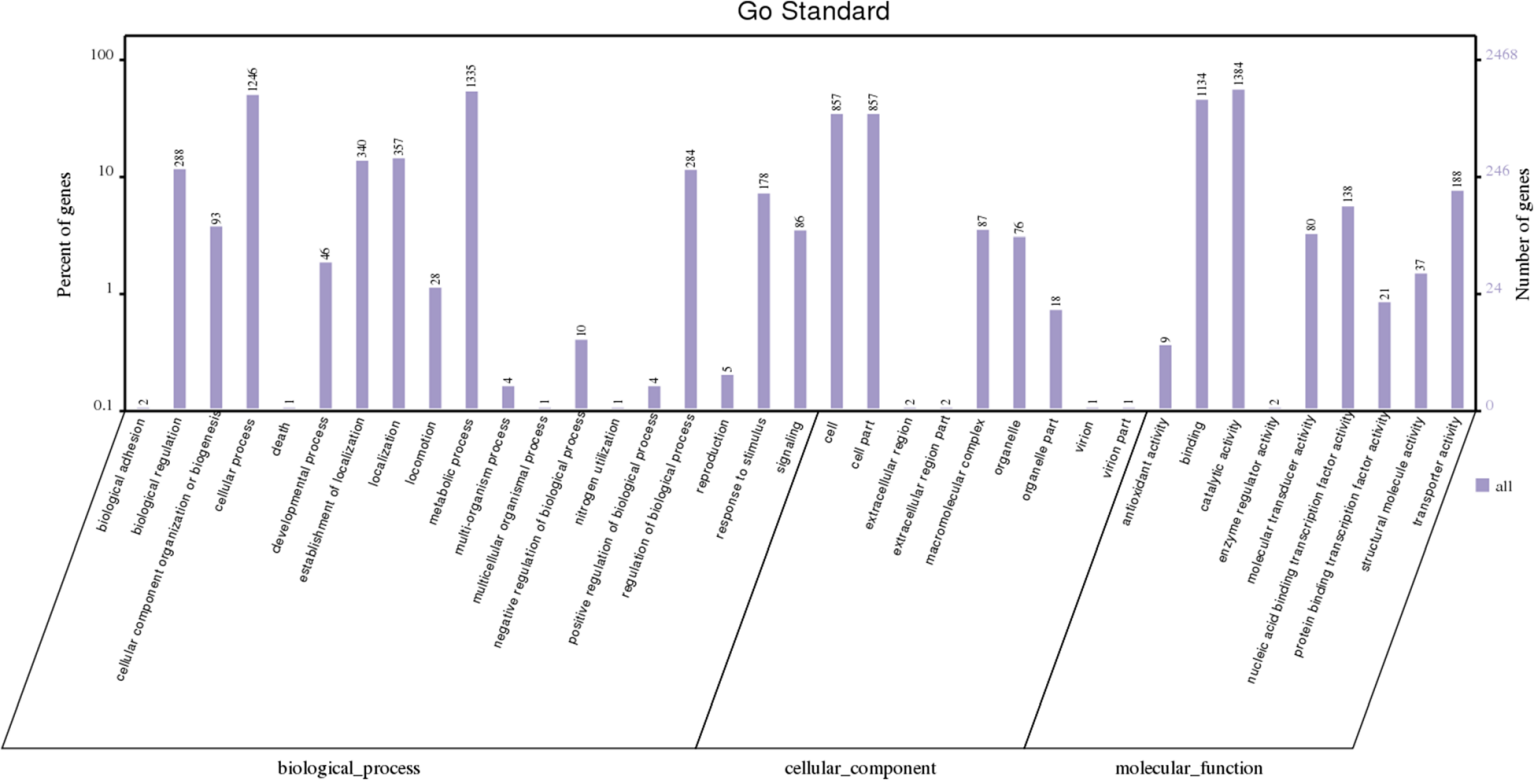
GO annotation of protein-coding genes

**Fig. 5:**
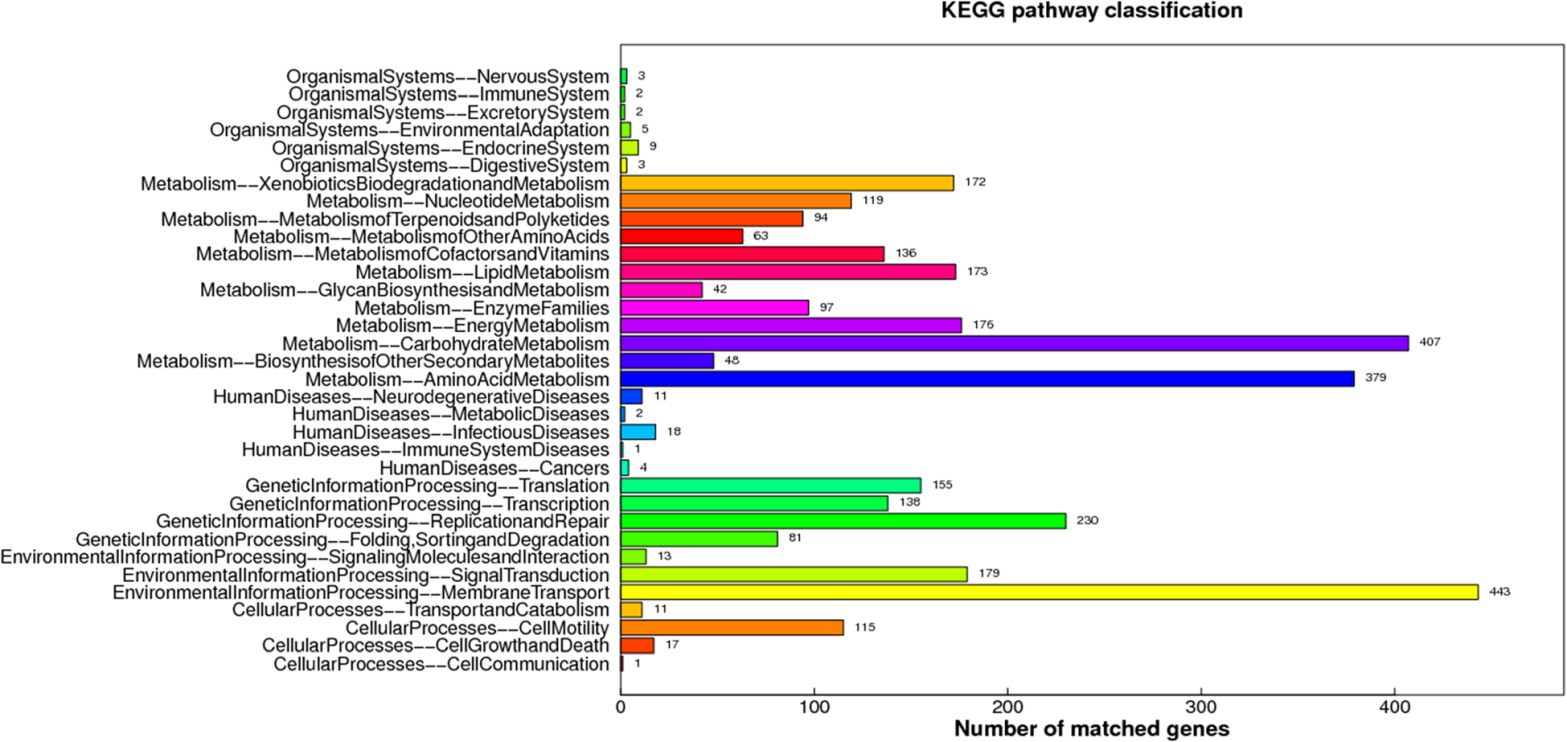
KEGG annotation of protein-coding genes

Protein coding genes were mainly classified into 3 parts based on their functions by GO analysis (Fig. 4). 1901, 2993 and 4309 genes participated in cellular component, molecular function and biological process, respectively. The metabolic pathway analysis using KEGG annotation showed that the majority of protein coding genes participated in metabolism, genetic information processing, environmental information processing and cellular processes (Fig. 5). 154 metabolic pathways were found using KEGG orthology, including glycolysis, TCA cycle and pentose phosphate pathways, fructose, mannose and galactose metabolisms pathways, fatty acid biosynthesis and metabolism pathways, ubiquinone and other terpenoid-uquinoid synthesis pathways, bacterial chemotaxis, biosynthsis of siderophore group nonribosomal peptides, antibiotic biosynthesis (tetracycline, penicillin and cephalosporin, streptomycin, novobiocin and vancomycin) as well as noxious substance degradation pathways (caprolactam, atrazine, ethylbenzene, toluene, polycyclic aromatic hydrocarbon, chloroalkane and chloroalkene, bisphenol, naphthalene, aminobenzoate, limonene and pinene), and so on.

Genome similarity was detected based on Mummer blast by comparing the genome sequence of strain TF28 with the type strain *B. amyloliquefaciens subsp. plantarum* FZB42^T^at amino acid level [21]. The results showed that genome similarity of *B. amyloliquefaciens* TF28 and *B. amyloliquefaciens subsp. plantarum* FZB42^T^ reached 98.69 %. Core-pan gene was also determined based on NCBI blast and Muscle analysis [22]. 201 strain-specific genes for *B. amyloliquefaciens* TF28 was observed, which may contribute to species-specific features of this bacterium. Among them, 83 genes are classified into 17 COG functional categories major belonging to carbohydrate transport and metabolism (6.97 %), general function prediction only (4.48 %), defense mechanisms (4.48 %), signal transduction mechanisms (3.48 %), amino acid transport and metabolism (3.98 %). The remaining 116 unique genes (57.71 %) are not classified into any COG categories (Table 5). Comparative genome analysis revealed that *B. amyloliquefaciens* TF28 possessed the giant gene clusters for non-ribosomal synthesis of the polyketides difficidin (TH57_02955-TH57_03045) and bacillaene (TH57_05575-TH57_05655), the antifungal lipopetides surfactin (TH57_12375-TH57_12430), plipastatin (TH57_04780-TH57_04835), mycosubtilin (TH57-04955-TH57-04980), bacilysin (TH57_15685-TH57_15710) and bacillibactin (TH57_05755-TH57_05800) (Additional file 2: Table S2). The size of these gene clusters accounted for 6.8 % of genome, which was smaller than that of strain FZB42^T^(8.9 %) [23]. Mycosubtilin and plipastatin synthesis gene clusters were only observed in strain TF28. These gene clusters produce the secondary metabolites like NRPSs, PKS, and peptide antibiotics usually displaying antifungal and antibacterial activities [23–25]. The finding of these gene clusters revealed that strain TF28 possessed a high potential to biocontrol. In addition, sporulation genes, *spo0ABFJ*(TH57_02695,01435,16015and14190),*spoVABCDEFKSMRT*(TH57_03250,03255,03260,03265,03270,03275,03280),*SpoIIBPMERDQSASB*(TH57_05470,00570,06315,09520,13850)*,spoIIIABCDEFGH*(TH57_01370,02065,03200,07810,07815,13800,16095,16205and16305)*,coaX*(TH57_01485)*,YtrIH*(TH57_00725,009730),*ylbJB*(TH57_06685),*ydcC*(TH57_11735),*ydhD*(TH57_07580),*cse15*(TH57_07135)*, yunB*(TH57_18555) and motility genes, *motAB* (TH57_074157, 07420) and *swrABC* (TH57_16980,05970 and 10760), were found in the genome.

**Table 5:** Number of strain-specific genes with general COG functional categories

Code	Value	%age	Description
J	2	0.99	Translation,ribosomal structure and biogenesis
A	0	0	RNA processing and modification
K	5	2.49	Transcription
L	4	1.99	Replication, recombination and repair
D	5	2.49	Cell cycle control, Cell division, chromosome partitioning
V	9	4.48	Defense mechanisms
T	7	3.48	Signal transduction mechanisms
N	1	0.49	Cell motility
C	3	1.49	Energy production and conversion
G	14	6.97	Carbohydrate transport and metabolism
E	8	3.98	Amino acid transport and metabolism
F	4	1.99	Nucleotide transport and metabolism
H	6	2.99	Coenzyme transport and metabolism
I	1	0.49	Lipid transport and metabolism
P	1	0.49	Inorganic ion transport and metabolism
Q	1	0.49	Secondary metabolites biosynthesis, transport and catabolism
R	9	4.48	General function prediction only
S	3	1.49	Function unknown
-	116	57.71	Not in COGs

**Fig. 6:**
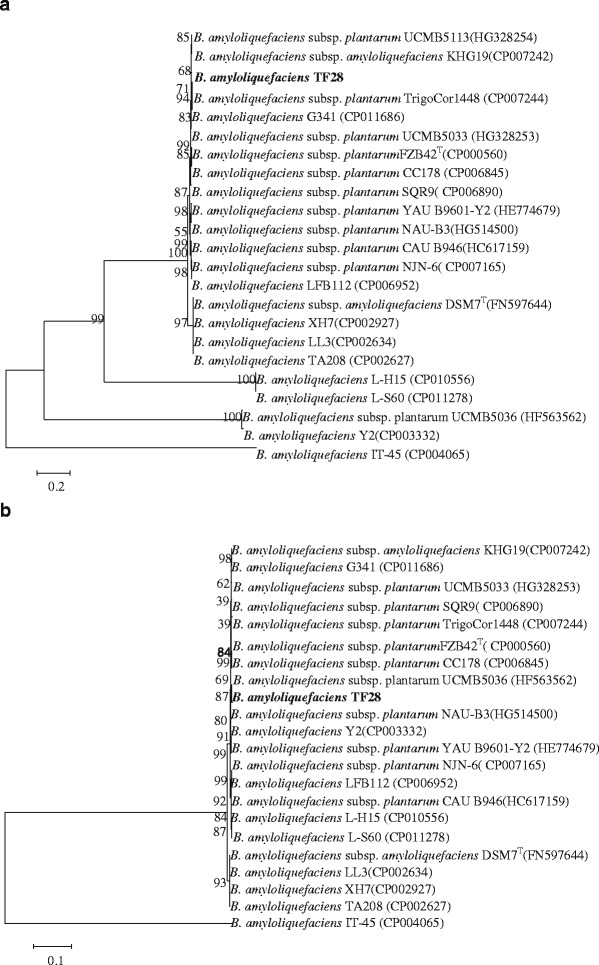
Phylogenetic trees based on *gyrA*(**a**) and *rpoB*(**b**). The GenBank accession numbers are shown in parentheses. Sequences were aligned using CLUSTALW, and phylogenetic inferences were constructed using the neighbor-joining method within the MEGA 5.10 software. Numbers at the nodes represent percentages of bootstrap values obtained by repeating the analysis 1000 times to generate a majority consensus tree. The scale bar indicates 0.2 (*gyr*A) and 0.1 (*rpoB*) nucleotide change per nucleotide position, respectively

Comparative genomic analysis of *B. amyloliquefaciens* TF28 and other 22 strains of *B. amyloliquefaciens* possessing complete genomic sequences indicated that the genome size of the strain TF28 was somewhat bigger than that of *B. amyloliquefaciens subsp. plantarum* FZB42^T^ and *B. amyloliquefaciens* subsp. *amyloliquefaciens*DSM7^T^. Three strains, *B. amyloliquefaciens* IT-45*,**B. amyloliquefaciens* NAU-B3 and *B. amyloliquefaciens* TF28, possessed CRISPR domains by CRISPR Finder on line (Additional file 3: Table S3, Additional file 4: Table S4, Additional file 5: Table S5, Additional file 6: Table S6). *B. amyloliquefaciens* TF28 possessed 3 CRISPR domains. The CRISPR length is 422 bp with 81 bp direct repeat (DR) sequences be separated by 5 spacers. No CRISPR associated gene was observed due to the incomplete genome sequence. *B. amyloliquefaciens* NAU-B3 had 1 CRISPR domains. The CRISPR length is 67 bp with 26 bp DR sequences be separated by 1 spacer. *B. amyloliquefaciens* IT-45 had 2 CRISPR domains. The CRISPR length is 129 bp with 37 bp DR sequences be separated by 1 spacer. The full-length sequence of protein-coding gene, DNA gyrase subunit A (*gyrA*) and RNA polymerase subunit B (*rpoB*) derived from 22 strains of *B. amyloliquefaciens*, were chosen to phylogenetic analysis. The neighbor-joining (NJ) phylogenetic tree revealed that strain TF28 with most of *B. amyloliquefaciens subsp. plantarum* clustered into the same group, which is distinct from the type strain *B. amyloliquefaciens* subsp. *amyloliquefaciens*DSM 7^T^(Fig. 6).

## Conclusions

In this study, we characterized the genome of *B. amyloliquefaciens* TF28 isolated from soybean root. Strain TF28 was classified as *B. amyloliquefaciens subsp. plantarum* on comparative analysis of 16S rRNA sequence, DNA gyrase subunit A (*gyrA*) and RNA polymerase subunit B (*rpoB*) gene sequences. The genome of strain TF28 has the giant gene clusters that are linked with biocontrol, including non-ribosomal synthesis of the polyketides difficidin and bacillaene, the antifungal lipopetides surfactin, plipastatin, mycosubtilin, bacilysin and bacillibactin. Mycosubtilin and plipastatin synthesis gene clusters were only observed in strain TF28. Ubiquinone and other terpenoid-uquinoid synthesis, bacterial chemotaxis, biosynthsis of siderophore group nonribosomal peptides, antibiotic biosynthesis and noxious substance degradation pathways were found which reflected a high capacity of strain TF28 to promote plant growth, inhibit pathogens and support environment fitness. 201 specific genes are found in strain TF28 which provides information for further analysis of the strain function. The availability of the genome provides insights to better understand the biocontrol mechanisms and facilitate the utilization of the strain in the future.

## Additional files

Additional file 1: Table S1. Strain ID Summary (DOCX 16 kb) Additional file 2: Table S2. Gene clusters of secondary metabolites synthesis in *B.amyloliquefaciens* TF28 and *B.amyloliquefaciens* subsp. plantarum FZB42^T^ (DOCX 13 kb) Additional file 3: Table S3. General feature of genome from 22 strains of *B. amyloliquefaciens* (DOCX 17 kb) Additional file 4: Table S4. GenBank Accession Summary (DOCX 15 kb) Additional file 5: Table S5. Scientific Name Summary (DOCX 12 kb) Additional file 6: Table S6. Reference Search Summary (DOCX 12 kb) Additional file 7: Table S7. Annotation Summary (DOCX 16 kb)

## Abbreviations

*gyrA*: DNA gyrase subunit A
*rpoB*: RNA polymerase subunit B
TCA: Tricarboxylic acid cycle
