# Supplementary material for: Whole genome shotgun sequence of Bacillus amyloliquefaciens TF28, a biocontrol entophytic bacterium
Source: Stand Genomic Sci. 2016 Sep 21;11:73. doi: 10.1186/s40793-016-0182-6 (PMC5031281; doi:10.1186/s40793-016-0182-6)
Supplement: Additional file 1: Table S1. — Strain ID Summary (DOCX 16 kb) [file 40793_2016_182_MOESM1_ESM.docx]

**Table S1:** Strain ID Summary

| **Strain ID** | **Summary** |
| --- | --- |
| LMG 22476^T^ | Collection Code: LMG  Collection Name: Belgian Coordinated Collections of Microorganisms/ LMG Bacteria Collection  Institution: LMG (Belgian Coordinated Collections of Microorganisms/ LMG Bacteria Collection) - Belgium  Strain ID: LMG 22476^T^ |
| DSM 7^T^ | Collection Code: DSM  Collection Name: Deutsche Sammlung von Mikroorganismen und Zellkulturen GmbH  Institution: DSM (Deutsche Sammlung von Mikroorganismen und Zellkulturen GmbH) - Deutschland  Strain ID: DSM 7^T^  External link:  <http://www.dsmz.de/catalogues/details/culture/DSM-7.html> |
| FZB42^T^ | Collection Code: DSM  Collection Name: Deutsche Sammlung von Mikroorganismen und Zellkulturen GmbH  Institution: DSM (Deutsche Sammlung von Mikroorganismen und Zellkulturen GmbH) - Deutschland  Strain ID: DSM23117^T^  External link:  <http://www.dsmz.de/catalogues/details/culture/DSM-23117.html> |
| NBRC 15718^T^ | Collection Code: NBRC  Collection Name: NITE Biological Resource Center  Institution: NBRC (NITE Biological Resource Center) - Japan  Strain ID: NBRC 15718^T^  External link:  <http://www.nbrc.nite.go.jp/NBRC2/NBRCCatalogueDetailServlet?ID=NBRC&CAT=15718> |
| NBRC 101239^T^ | Collection Code: NBRC  Collection Name: NITE Biological Resource Center  Institution: NBRC (NITE Biological Resource Center) - Japan  Strain ID: NBRC 101239^T^  External link:  <http://www.nbrc.nite.go.jp/NBRC2/NBRCCatalogueDetailServlet?ID=NBRC&CAT=101239> |
| DSM 10^T^ | Collection Code: DSM  Collection Name: Deutsche Sammlung von Mikroorganismen und Zellkulturen GmbH  Institution: DSM (Deutsche Sammlung von Mikroorganismen und Zellkulturen GmbH) - Deutschland  Strain ID: DSM 10^T^  External link:  <http://www.dsmz.de/catalogues/details/culture/DSM-10.html> |
| DSM 11031^T^ | Collection Code: DSM  Collection Name: Deutsche Sammlung von Mikroorganismen und Zellkulturen GmbH  Institution: DSM (Deutsche Sammlung von Mikroorganismen und Zellkulturen GmbH) - Deutschland  Strain ID: DSM 11031T  External link:  <http://www.dsmz.de/catalogues/details/culture/DSM-11031.html> |
| NBRC 15539^T^ | Collection Code: NBRC  Collection Name: NITE Biological Resource Center  Institution: NBRC (NITE Biological Resource Center) - Japan  Strain ID: NBRC 15539^T^  External link:  <http://www.nbrc.nite.go.jp/NBRC2/NBRCCatalogueDetailServlet?ID=NBRC&CAT=15539> |
| CR-95^T^ | Collection Code: LMG  Collection Name: Belgian Coordinated Collections of Microorganisms/ LMG Bacteria Collection  Institution: LMG (Belgian Coordinated Collections of Microorganisms/ LMG Bacteria Collection) - Belgium  Strain ID: LMG 22476^T^ |
| BGSC 3A28^T^ | Collection Code: NRRL  Collection Name: Agricultural Research Service Culture Collection, National Center for Agricultural Utilization Research, US Department of Agriculture, Peoria, IL, USA.  Institution: NRRL-American  Strain ID: NRRL B-23052^T^ |
| 10b^T^ | Collection Code: ATCC  Collection Name: American Type Culture Collection, Manassas, VA, USA  Institution: ATCC-American  Strain ID: [ATCC BAA-819](http://www.atcc.org/products/all/BAA-819.aspx)^T^ |
| PD-A10^T^ | Collection Code: KCTC  Collection Name: Korean Collection for Type Cultures, Genetic Resources Center, Institution: Korea Research Institute of Bioscience and Biotechnology, Taejon, Republic of Korea.  Strain ID: KCTC 13613^T^ |
| CBMB205^T^ | Collection Code: KACC  Collection Name: Korean Agricultural Culture Collection.  Institution: National Institute of Agricultural Biotechnology, Rural Development Administration, Suwon, Republic of Korea.  Strain ID: KACC 13105^T^ |
| TF28 | Collection Code: CGMCC  Collection Name: China General Microbiological Culture Collection Center.  Institution: China General Microbiological Culture Collection Center, Institute of Microbiology, Chinese Academy of Sciences, Beijing, China  Strain ID: CGMCC No.4038 |
| 168^T^ | Collection Code: ATCC  Collection Name: American Type Culture Collection, Manassas, VA, USA  Institution: ATCC-American  Strain ID: ATCC168^T^ |
