# Supplementary material for: Whole genome shotgun sequence of Bacillus amyloliquefaciens TF28, a biocontrol entophytic bacterium
Source: Stand Genomic Sci. 2016 Sep 21;11:73. doi: 10.1186/s40793-016-0182-6 (PMC5031281; doi:10.1186/s40793-016-0182-6)
Supplement: Additional file 2: Table S2. — Gene clusters of secondary metabolites synthesis in B.amyloliquefaciens TF28 and B.amyloliquefaciens subsp. plantarum FZB42T (DOCX 13 kb) [file 40793_2016_182_MOESM2_ESM.docx]

**Table S2.** Gene clusters of secondary metabolites synthesis in ***B.amyloliquefaciens*** TF28 and ***B.amyloliquefaciens*** subsp. *plantarum* FZB42^T^[37]

| **Compound** | **Enzyme** | **TF28** | **Size**  **(kb)** | **FZB42^T^** | **Size** |
| --- | --- | --- | --- | --- | --- |
| Surfactin | NRPS | *srfABCD* | 26.0 | *srfABCD,aat,334,*  *ycx,cycD,sfp, yczE* | 32.0 |
| Plipastatin | NRPS | *ppsABCDE* | 34.0 | - | - |
| Mycosubtilin | NRPS | *mycABCD* | 36.9 | - | - |
| Bacilysin | NRPS | *bacABCDE,ywfG* | 6.1 | *bacABCDE, ywfG* | 6.9 |
| Bacillaene | NRPS/  PKS | *pksGHIJLMNRS,acpK,baeBCDE* | 72.2 | *baeGHIJLMNRS,*  *acpK,*  *baeBCDE* | 74.3 |
| Difficidin | PKS | *pksGIM* | 68.8 | *dfnAYXBCDEFGHIJKLM* | 71.1 |
| Bacillibactin | NRPS | *ymfDFHIJKM* | 11.7 | *dhbABCDEF* | 12.8 |
