# Supplementary material for: Whole genome shotgun sequence of Bacillus amyloliquefaciens TF28, a biocontrol entophytic bacterium
Source: Stand Genomic Sci. 2016 Sep 21;11:73. doi: 10.1186/s40793-016-0182-6 (PMC5031281; doi:10.1186/s40793-016-0182-6)
Supplement: Additional file 3: Table S3. — General feature of genome from 22 strains of B. amyloliquefaciens (DOCX 17 kb) [file 40793_2016_182_MOESM3_ESM.docx]

**Supplemental table 3. General feature of** genome from **22** **strains of** ***B. amyloliquefaciens***

| **Strains** | **Size (Mb)** | **G+C %** | **CDS_S_** | **rRNA** | **tRNA** | **CRISPR** | **GenBank No.** | **NCBI BioProject number** |
| --- | --- | --- | --- | --- | --- | --- | --- | --- |
| ***B.amyloliquefaciens*** subsp. *plantarum* FZB42^T^ | 3.91 | 46.50 | 3627 | 29 | 89 | 0 | CP000560 | PRJAN13403 |
| ***B.amyloliquefaciens*** subsp. ***amyloliquefaciens*** DSM7^T^ | 3.98 | 46.00 | 3811 | 30 | 94 | 0 | FN597644 | PRJEA41719 |
| ***B.amyloliquefaciens*** subsp. *plantarum* Trigour1448 | 3.95 | 46.50 | 3683 | 24 | 77 | 0 | CP007244 | PRJAN234772 |
| ***B.amyloliquefaciens*** subsp. *plantarum* SQR9 | 4.12 | 46.10 | 3841 | 21 | 72 | 0 | CP006890 | PRJAN227504 |
| ***B.amyloliquefaciens*** subsp. *plantarum* UCMB5113 | 3.88 | 46.70 | 3612 | 29 | 89 | 0 | HG328254 | PRJEB1418 |
| ***B.amyloliquefaciens*** subsp. *plantarum* CAUB946 | 4.10 | 46.50 | 3745 | 30 | 95 | 0 | HC617159 | PRJAN76795 |
| ***B.amyloliquefaciens*** subsp. *plantarum* UCMB5036 | 3.91 | 46.60 | 3634 | 29 | 89 | 0 | HF563562 | PRJEB1155 |
| ***B.amyloliquefaciens*** subsp. *plantarum* UCMB5033 | 4.07 | 46.20 | 3828 | 30 | 86 | 0 | HG328253 | PRJEB3961 |
| ***B.amyloliquefaciens*** Y2 | 4.23 | 45.90 | 3983 | 29 | 87 | 0 | CP003332 | PRJAN78839 |
| ***B.amyloliquefaciens*** LL3 | 4.00 | 45.69 | 3887 | 22 | 72 | 0 | CP002634 | PRJAN64659 |
| ***B.amyloliquefaciens*** XH7 | 3.93 | 45.80 | 3846 | 21 | 75 | 0 | CP002927 | PRJAN67079 |
| ***B.amyloliquefaciens*** subsp. *plantarum* YAUB9601-Y2 | 4.24 | 45.90 | 3987 | 30 | 91 | 0 | HE774679 | PRJEA86121 |
| ***B.amyloliquefaciens*** subsp. *plantarum* NAU-B3 | 4.20 | 46.00 | 3888 | 30 | 91 | 1 | HG514500 | PRJEB4476 |
| ***B.amyloliquefaciens*** subsp. *plantarum* NJN-6 | 4.05 | 46.60 | 3785 | 25 | 81 | 0 | CP007165 | PRJAN236411 |
| ***B.amyloliquefaciens*** IT-45 | 3.93 | 46.60 | 3670 | 30 | 95 | 2 | CP004119 | PRJAN73591 |
| ***B.amyloliquefaciens*** TA208 | 3.94 | 45.8 | 3847 | 18 | 70 | 0 | CP002627 | PRJAN64581 |
| ***B.amyloliquefaciens*** CC178 | 3.92 | 46.50 | 3641 | 27 | 86 | 0 | CP006845 | PRJAN224754 |
| ***B.amyloliquefaciens*** LFB112 | 3.94 | 46.70 | 3637 | 30 | 94 | 0 | CP006952 | PRJAN225659 |
| ***B.amyloliquefaciens*** L-H15 | 3.91 | 46.70 | 3615 | 26 | 83 | 0 | CP0101556 | PRJAN271047 |
| ***B.amyloliquefaciens*** subsp. ***amyloliquefaciens*** KHG19 | 3.95 | 46.60 | 3658 | 28 | 89 | 0 | CP007242 | PRJAN184846 |
| ***B.amyloliquefaciens*** L-S60 | 3.90 | 46.70 | 3611 | 26 | 91 | 0 | CP0011278 | PRJAN280801 |
| ***B.amyloliquefaciens*** G341 | 4.01 | 46.50 | 3743 | 30 | 95 | 0 | CP011686 | PRJAN284876 |
| ***B.amyloliquefaciens*** TF28 | 3.97 | 46.38 | 3571 | 7 | 63 | 3 | JUDU00000000 | PRJAN268537 |
