# Supplementary material for: Whole genome shotgun sequence of Bacillus amyloliquefaciens TF28, a biocontrol entophytic bacterium
Source: Stand Genomic Sci. 2016 Sep 21;11:73. doi: 10.1186/s40793-016-0182-6 (PMC5031281; doi:10.1186/s40793-016-0182-6)
Supplement: Additional file 4: Table S4. — GenBank Accession Summary (DOCX 15 kb) [file 40793_2016_182_MOESM4_ESM.docx]

**Table S4:** GenBank Accession Summary

| **GenBank Accession** | **Summary** |
| --- | --- |
| HG514500 | HG514500.1 is a bacterial sequences record containing circular, double-stranded DNA (8,438 bases) from Bacillus methylotrophicus strain NAU-B3. The record was created on October 3, 2013 and last updated February 27, 2015. It contains 11 features, including 5 genes with 2 distinct annotations, 5 DNA coding regions. |
| HE617159 | HE617159.1 is a bacterial sequences record containing circular, double-stranded DNA (4,019,861 bases) from Bacillus methylotrophicus CAU B946 strain CAU B946. The record was created on January 9, 2012 and last updated February 27, 2015. It contains 7,897 features, including 3948 genes with 3512 distinct annotations, 3823 DNA coding regions, 30 rRNA features (16S ribosomal RNA, 5S ribosomal RNA and 23S ribosomal RNA) and 95 tRNA features coding for 20 distinct amino acids. |
| CP004119 | CP004119.1 is a bacterial sequences record containing circular, double-stranded DNA (8,009 bases) from Bacillus amyloliquefaciens strain IT-45. The record was created on February 13, 2013 and last updated January 30, 2014. It contains 19 features, including, 9 DNA coding regions. |
| HF563562 | HF563562.1 is a bacterial sequences record containing circular, double-stranded DNA (3,910,324 bases) from Bacillus methylotrophicus strain UCMB5036. The record was created on February 21, 2013 and last updated February 27, 2015. It contains 7,685 features, including 3842 genes with 3333 distinct annotations, 3660 DNA coding regions, 29 rRNA features (ribosomal RNA 5S ribosomal RNA, ribosomal RNA 23S ribosomal RNA and ribosomal RNA 16S ribosomal RNA) and 89 tRNA features coding for 20 distinct amino acids. |
| CP006845 | CP006845.1 is a bacterial sequences record containing circular, double-stranded DNA (3,916,828 bases) from Bacillus amyloliquefaciens strain CC178. The record was created on November 1, 2013 and last updated January 29, 2015. It contains 8,138 features, including, 3950 DNA coding regions, 27 rRNA features (16S ribosomal RNA, 5S ribosomal RNA and 23S ribosomal RNA) and 86 tRNA features coding for 20 distinct amino acids. |
| HE774679 | HE774679.1 is a bacterial sequences record containing circular, double-stranded DNA (4,242,774 bases) from Bacillus methylotrophicus YAU B9601-Y2 strain YAU B9601-Y2. The record was created on March 15, 2012 and last updated February 27, 2015. It contains 8,221 features, including 4110 genes with 3580 distinct annotations, 3989 DNA coding regions, 30 rRNA features (16S ribosomal RNA, 5S ribosomal RNA and 23S ribosomal RNA) and 91 tRNA features coding for 20 distinct amino acids. |
| CP003332 | CP003332.1 is a bacterial sequences record containing circular, double-stranded DNA (4,238,624 bases) from Bacillus amyloliquefaciens strain Y2. The record was created on May 11, 2012 and last updated January 30, 2014. It contains 8,705 features, including 4352 genes with 2633 distinct annotations, 4238 DNA coding regions, 29 rRNA features (16S ribosomal RNA, 5S ribosomal RNA and 23S ribosomal RNA) and 85 tRNA features coding for 20 distinct amino acids. |
| CP006952 | CP006952.1 is a bacterial sequences record containing circular, double-stranded DNA (3,942,754 bases) from Bacillus amyloliquefaciens strain LFB112. The record was created on December 16, 2013 and last updated February 14, 2014. It contains 8,028 features, including 4023 genes with 126 distinct annotations, 3859 DNA coding regions, 30 rRNA features (16S ribosomal RNA, 5S ribosomal RNA and 23S ribosomal RNA) and 94 tRNA features coding for 20 distinct amino acids. |
| CP007165 | CP007165.1 is a bacterial sequences record containing circular, double-stranded DNA (4,052,546 bases) from Bacillus methylotrophicus strain NJN-6. The record was created on April 20, 2015 and last updated July 20, 2015. It contains 8,004 features, including 3999 genes with 1160 distinct annotations, 3894 DNA coding regions, 24 rRNA features (16S ribosomal RNA, 5S ribosomal RNA and 23S ribosomal RNA) and 81 tRNA features coding for 20 distinct amino acids. |
| HG328253 | HG328253.1 is a bacterial sequences record containing circular, double-stranded DNA (4,071,167 bases) from Bacillus methylotrophicus UCMB5033 strain UCMB-5033. The record was created on August 9, 2013 and last updated February 27, 2015. It contains 8,191 features, including 4095 genes with 3279 distinct annotations, 3912 DNA coding regions, 30 rRNA features (ribosomal RNA 5S ribosomal RNA, ribosomal RNA 23S ribosomal RNA and ribosomal RNA 16S ribosomal RNA) and 86 tRNA features coding for 20 distinct amino acids. |
| CP002627 | CP002627.1 is a bacterial sequences record containing circular, double-stranded DNA (3,937,511 bases) from Bacillus amyloliquefaciens strain TA208. The record was created on April 8, 2011 and last updated January 31, 2014. It contains 8,373 features, including 4177 genes with 3231 distinct annotations, 4089 DNA coding regions, 18 rRNA features (16S ribosomal RNA, 5S ribosomal RNA and 23S ribosomal RNA) and 70 tRNA features coding for 20 distinct amino acids. |
| CP007242 | CP007242.1 is a bacterial sequences record containing circular, double-stranded DNA (3,953,361 bases) from Bacillus amyloliquefaciens strain KHG19. The record was created on February 10, 2015. It contains 7,612 features, including 3820 genes with 857 distinct annotations, 3675 DNA coding regions, 28 rRNA features (16S ribosomal RNA, 5S ribosomal RNA and 23S ribosomal RNA) and 88 tRNA features coding for 20 distinct amino acids. |
| FN597644 | FN597644.1 is a bacterial sequences record containing circular, double-stranded DNA (3,980,199 bases) from Bacillus amyloliquefaciens DSM 7 strain DSM 7. The record was created on September 23, 2010 and last updated February 27, 2015. It contains 8,093 features, including 4046 genes with 3676 distinct annotations, 3922 DNA coding regions, 30 rRNA features (16S ribosomal RNA, 5S ribosomal RNA and 23S ribosomal RNA) and 94 tRNA features coding for 20 distinct amino acids. |
| CP002927 | CP002927.1 is a bacterial sequences record containing circular, double-stranded DNA (3,939,203 bases) from Bacillus amyloliquefaciens strain XH7. The record was created on July 27, 2011 and last updated January 31, 2014. It contains 8,593 features, including 4286 genes with 3122 distinct annotations, 4190 DNA coding regions, 21 rRNA features (16S ribosomal RNA, 5S ribosomal RNA and 23S ribosomal RNA) and 75 tRNA features coding for 20 distinct amino acids. |
| PRJNA268537 | BioProject PRJNA268537: http://www.ncbi.nlm.nih.gov/bioproject/PRJNA268537 |
| CP002634 | CP002634.1 is a bacterial sequences record containing circular, double-stranded DNA (3,995,227 bases) from Bacillus amyloliquefaciens strain LL3. The record was created on April 14, 2011 and last updated January 30, 2014. It contains 8,660 features, including 4346 genes with 2650 distinct annotations, 4219 DNA coding regions, 22 rRNA features (16S ribosomal RNA, 5S ribosomal RNA and 23S ribosomal RNA) and 72 tRNA features coding for 20 distinct amino acids. |
| CP000560 | CP000560.1 is a bacterial sequences record containing circular, double-stranded DNA (3,918,589 bases) from Bacillus amyloliquefaciens subsp. plantarum str. strain FZB42. The record was created on August 1, 2007 and last updated February 24, 2014. It contains 7,625 features, including 3812 genes with 3223 distinct annotations, 3693 DNA coding regions, 30 rRNA features (16S ribosomal RNA, 5S ribosomal RNA and 23S ribosomal RNA) and 86 tRNA features coding for 20 distinct amino acids. |
| CP011686 | CP011686.1 is a bacterial sequences record containing circular, double-stranded DNA (4,009,746 bases) from Bacillus amyloliquefaciens strain G341. The record was created on June 10, 2015. It contains 8,193 features, including, 3953 DNA coding regions, 30 rRNA features (16S ribosomal RNA, 5S ribosomal RNA and 23S ribosomal RNA) and 95 tRNA features coding for 20 distinct amino acids. |
| CP006890 | CP006890.1 is a bacterial sequences record containing circular, double-stranded DNA (4,117,023 bases) from Bacillus methylotrophicus strain SQR9. The record was created on May 8, 2014 and last updated September 9, 2015. It contains 8,343 features, including 4171 genes with 2453 distinct annotations, 4078 DNA coding regions, 21 rRNA features (16S ribosomal RNA, 5S ribosomal RNA and 23S ribosomal RNA) and 72 tRNA features coding for 20 distinct amino acids. |
| CP007244 | CP007244.1 is a bacterial sequences record containing circular, double-stranded DNA (3,957,904 bases) from Bacillus methylotrophicus strain TrigoCor1448. The record was created on February 28, 2014. It contains 7,673 features, including 3857 genes with 102 distinct annotations, 3683 DNA coding regions, 24 rRNA features (16S ribosomal RNA, 5S ribosomal RNA and 23S ribosomal RNA) and 77 tRNA features coding for 20 distinct amino acids. |
| HG328254 | HG328254.1 is a bacterial sequences record containing circular, double-stranded DNA (3,889,532 bases) from Bacillus methylotrophicus strain UCMB5113. The record was created on August 9, 2013 and last updated February 27, 2015. It contains 7,709 features, including 3854 genes with 3271 distinct annotations, 3672 DNA coding regions, 29 rRNA features (ribosomal RNA 5S ribosomal RNA, ribosomal RNA 23S ribosomal RNA and ribosomal RNA 16S ribosomal RNA) and 89 tRNA features coding for 20 distinct amino acids. |
| JUDU00000000 | JUDU00000000.1 is a bacterial sequences record containing linear, double-stranded DNA (182 bases) from Bacillus amyloliquefaciens strain TF28. The record was created on January 12, 2015 and last updated June 30, 2015. It contains 1 feature. |
