# Supplementary material for: Whole genome shotgun sequence of Bacillus amyloliquefaciens TF28, a biocontrol entophytic bacterium
Source: Stand Genomic Sci. 2016 Sep 21;11:73. doi: 10.1186/s40793-016-0182-6 (PMC5031281; doi:10.1186/s40793-016-0182-6)
Supplement: Additional file 5: Table S5. — Scientific Name Summary (DOCX 12 kb) [file 40793_2016_182_MOESM5_ESM.docx]

**Table S5:** Scientific Name Summary

| **Name** | **Summary** |
| --- | --- |
| This | NCBI Taxonomy ID: 169495  Links:  <http://www.ncbi.nlm.nih.gov/Taxonomy/Browser/wwwtax.cgi?lvl=0&id=169495> |
| Meta | NCBI Taxonomy ID: 94025  Links:  <http://www.ncbi.nlm.nih.gov/Taxonomy/Browser/wwwtax.cgi?lvl=0&id=94025> |
| Paris | NCBI Taxonomy ID: 49669  Links:  <http://www.ncbi.nlm.nih.gov/Taxonomy/Browser/wwwtax.cgi?lvl=0&id=49669> |
| Eucarya | NCBI Taxonomy ID: 2759  Links:  <http://www.ncbi.nlm.nih.gov/Taxonomy/Browser/wwwtax.cgi?lvl=0&id=2759> |
| Kluyve romyces lactis | NCBI Taxonomy ID: 28985  Links:  <http://www.ncbi.nlm.nih.gov/Taxonomy/Browser/wwwtax.cgi?lvl=0&id=28985> |
