# Supplementary material for: Whole genome shotgun sequence of Bacillus amyloliquefaciens TF28, a biocontrol entophytic bacterium
Source: Stand Genomic Sci. 2016 Sep 21;11:73. doi: 10.1186/s40793-016-0182-6 (PMC5031281; doi:10.1186/s40793-016-0182-6)
Supplement: Additional file 6: Table S6. — Reference Search Summary (DOCX 12 kb) [file 40793_2016_182_MOESM6_ESM.docx]

**Table S6:** Reference Search Summary

| **Name** | **Occurence** |
| --- | --- |
| Stand Genomic Sci | 1 |
