# Supplementary material for: Whole genome shotgun sequence of Bacillus amyloliquefaciens TF28, a biocontrol entophytic bacterium
Source: Stand Genomic Sci. 2016 Sep 21;11:73. doi: 10.1186/s40793-016-0182-6 (PMC5031281; doi:10.1186/s40793-016-0182-6)
Supplement: Additional file 7: Table S7. — Annotation Summary (DOCX 16 kb) [file 40793_2016_182_MOESM7_ESM.docx]

**Table S7:** Annotation Summary

| **Name** | **Count** | **References** | **Comments** |
| --- | --- | --- | --- |
| *Bacillaceae* | 1 | FAMILY Bacillaceae:  V.B.D. Skerman, V. McGowan, P.H.A. Sneath: Approved Lists of Bacterial Names. International Journal of Systematic Bacteriology 1980, 30: 225-420.  A. Fischer: Untersuchungen über bakterien. Jahrbücher für Wissenschaftliche Botanik 1895, 27: 1-163. |  |
| *Bacillales* | 1 | ORDER Bacillales:  V.B.D. Skerman, V. McGowan, P.H.A. Sneath: Approved Lists of Bacterial Names. International Journal of Systematic Bacteriology 1980, 30: 225-420.  A.R. Prévot: Dictionnaire des Bactéries Pathogènes. Dictionnaire des Bactéries Pathogènes 1953, 1-692. |  |
| *Bacilli* | 2 | CLASS Firmibacteria:  List Editor: List of new names and new combinations previously effectively, but not validly, published. List no. 132. International Journal of Systematic and Evolutionary Microbiology 2010, 60: 469-472.  W. Ludwig, K.-H. Schleifer, W. B. Whitman: Class I. Bacilli class nov. Bergey's Manual of Systematic Bacteriology 2009, 3: 19-20. |  |
| *Bacillus* | 6 | GENUS Bacillus:  V.B.D. Skerman, V. McGowan, P.H.A. Sneath: Approved Lists of Bacterial Names. International Journal of Systematic Bacteriology 1980, 30: 225-420.  F. Cohn: Untersuchungen über Bakterien. Beiträge zur Biologie der Pflanzen 1872, 1: 127-224. |  |
| *Bacillus amyloliquefaciens* | 38 | SPECIES Bacillus amyloliquefaciens:  F.G. Priest, M. Goodfellow, L.A. Shute, R.C.W. Berkeley: Bacillus amyloliquefaciens sp. nov., nom. rev. International Journal of Systematic Bacteriology 1987, 37: 69-71. |  |
| *Bacillus amyloliquefaciens amyloliquefaciens* | 6 | SUBSPECIES Bacillus amyloliquefaciens:  R. Borriss, X.-H. Chen, C. Rueckert, J. Blom, A. Becker, B. Baumgarth, B. Fan, R. Pukall, P. Schumann, C. Spröer, H. Junge, J. Vater, A. Pühler, H.-P. Klenk: Relationship of Bacillus amyloliquefaciens clades associated with strains DSM 7T and FZB42T: a proposal for Bacillus amyloliquefaciens subsp. amyloliquefaciens subsp. nov. and Bacillus amyloliquefaciens subsp. plantarum subsp. nov. based on complete genome sequence comparisons. International Journal of Systematic and Evolutionary Microbiology 2011, 61: 1786-1801. |  |
| *Bacillus amyloliquefaciens plantarum* | 10 | SUBSPECIES Bacillus amyloliquefaciens plantarum:  R. Borriss, X.-H. Chen, C. Rueckert, J. Blom, A. Becker, B. Baumgarth, B. Fan, R. Pukall, P. Schumann, C. Spröer, H. Junge, J. Vater, A. Pühler, H.-P. Klenk: Relationship of Bacillus amyloliquefaciens clades associated with strains DSM 7T and FZB42T: a proposal for Bacillus amyloliquefaciens subsp. amyloliquefaciens subsp. nov. and Bacillus amyloliquefaciens subsp. plantarum subsp. nov. based on complete genome sequence comparisons. International Journal of Systematic and Evolutionary Microbiology 2011, 61: 1786-1801. |  |
| *Bacillus atrophaeus* | 1 | SPECIES Bacillus atrophaeus:  L.K. Nakamura: Taxonomic relationship of black-pigmented Bacillus subtilis strains and a proposal for Bacillus atrophaeus sp. nov. International Journal of Systematic Bacteriology 1989, 39: 295-300. |  |
| *Bacillus axarquiensis* | 1 | SPECIES Bacillus axarquiensis:  C. Ruiz-García, E. Quesada, F. Martínez-Checa, I. Llamas, M.C. Urdaci, V. Béjar: Bacillus axarquiensis sp. nov. and Bacillus malacitensis sp. nov., isolated from river-mouth sediments in southern Spain. International Journal of Systematic and Evolutionary Microbiology 2005, 55: 1279-1285. |  |
| *Bacillus malacitensis* | 1 | SPECIES Bacillus malacitensis:  C. Ruiz-García, E. Quesada, F. Martínez-Checa, I. Llamas, M.C. Urdaci, V. Béjar: Bacillus axarquiensis sp. nov. and Bacillus malacitensis sp. nov., isolated from river-mouth sediments in southern Spain. International Journal of Systematic and Evolutionary Microbiology 2005, 55: 1279-1285. |  |
| *Bacillus methylotrophicus* | 2 | SPECIES Bacillus methylotrophicus:  M. Madhaiyan, S. Poonguzhali, S.-W. Kwon, T.-M. Sa: Bacillus methylotrophicus sp. nov., a methanol-utilizing, plant-growth-promoting bacterium isolated from rice rhizosphere soil. International Journal of Systematic and Evolutionary Microbiology 2010, 60: 2490-2495. |  |
| *Bacillus mojavensis* | 1 | SPECIES Bacillus mojavensis:  M.S. Roberts, L.K. Nakamura, F.M. Cohan: Bacillus mojavensis sp. nov., distinguishable from Bacillus subtilis by sexual isolation, divergence in DNA sequence, and differences in fatty acid composition. International Journal of Systematic Bacteriology 1994, 44: 256-264. |  |
| *Bacillus siamensis* | 1 | SPECIES Bacillus siamensis:  P. Sumpavapol, L. Tongyonk, S. Tanasupawat, N. Chokesajjawatee, P. Luxananil, W. Visessanguan: Bacillus siamensis sp. nov., isolated from salted crab (poo-khem) in Thailand. International Journal of Systematic and Evolutionary Microbiology 2010, 60: 2364-2370. |  |
| *Bacillus subtilis* | 1 | SPECIES Bacillus subtilis:  V.B.D. Skerman, V. McGowan, P.H.A. Sneath: Approved Lists of Bacterial Names. International Journal of Systematic Bacteriology 1980, 30: 225-420.  F. Cohn: Untersuchungen über Bakterien. Beiträge zur Biologie der Pflanzen 1872, 1: 127-224. |  |
| *Bacillus subtilis inaquosorum* | 1 | SUBSPECIES Bacillus subtilis inaquosorum:  A.P. Rooney, N.P.J. Price, C. Ehrhardt, J.L. Swezey, J.D. Bannan: Phylogeny and molecular taxonomy of the Bacillus subtilis species complex and description of Bacillus subtilis subsp. inaquosorum subsp. nov. International Journal of Systematic and Evolutionary Microbiology 2009, 59: 2429-2436. |  |
| *Bacillus subtilis spizizenii* | 1 | SUBSPECIES Bacillus subtilis spizizenii:  L.K. Nakamura, M.S. Roberts, F.M. Cohan: Relationship of Bacillus subtilis clades associated with strains 168 and W23: a proposal for Bacillus subtilis subsp. subtilis subsp. nov. and Bacillus subtilis subsp. spizizenii subsp. nov. International Journal of Systematic Bacteriology 1999, 49: 1211-1215. |  |
| *Bacillus subtilis subtilis* | 1 | SUBSPECIES Bacillus subtilis:  L.K. Nakamura, M.S. Roberts, F.M. Cohan: Relationship of Bacillus subtilis clades associated with strains 168 and W23: a proposal for Bacillus subtilis subsp. subtilis subsp. nov. and Bacillus subtilis subsp. spizizenii subsp. nov. International Journal of Systematic Bacteriology 1999, 49: 1211-1215. |  |
| *Bacillus tequilensis* | 1 | SPECIES Bacillus tequilensis:  J.W. Gatson, B.F. Benz, C. Chandrasekaran, M. Satomi, K. Venkateswaran, M.E. Hart: Bacillus tequilensis sp. nov., isolated from a 2000-year-old Mexican shaft-tomb, is closely related to Bacillus subtilis. International Journal of Systematic and Evolutionary Microbiology 2006, 56: 1475-1484. |  |
| *Bacillus vallismortis* | 1 | SPECIES Bacillus vallismortis:  M.S. Roberts, L.K. Nakamura, F.M. Cohan: Bacillus vallismortis sp. nov., a close relative of Bacillus subtilis, isolated from soil in Death Valley, California. International Journal of Systematic Bacteriology 1996, 46: 470-475. |  |
| *Bacillus velezensis* | 1 | SPECIES Bacillus velezensis:  C. Ruiz-García, V. Béjar, F. Martínez-Checa, I. Llamas, E. Quesada: Bacillus velezensis sp. nov., a surfactant-producing bacterium isolated from the river Vélez in Málaga, southern Spain. International Journal of Systematic and Evolutionary Microbiology 2005, 55: 191-195. |  |
| *Bifido bacterium* | 1 | GENUS Bifidobacterium:  S. Orla-Jensen: Classification des bactéries lactiques. Le Lait 1924, 4: 468-474.  V.B.D. Skerman, V. McGowan, P.H.A. Sneath: Approved Lists of Bacterial Names. International Journal of Systematic Bacteriology 1980, 30: 225-420. |  |
| *Escherichia coli* | 1 | SPECIES Escherichia coli:  A. Castellani, A.J. Chalmers: Genus Escherichia Castellani and Chalmers, 1918. Manual of Tropical Medicine 1919, 941-943.  V.B.D. Skerman, V. McGowan, P.H.A. Sneath: Approved Lists of Bacterial Names. International Journal of Systematic Bacteriology 1980, 30: 225-420. |  |
| Firmicutes | 1 | PHYLUM Firmicutes:  N.E. Gibbons, R.G.E. Murray: Proposals Concerning the Higher Taxa of Bacteria. International Journal of Systematic Bacteriology 1978, 28: 1-6. |  |
